# Tractography-based Parcellation of the Human Middle Temporal Gyrus

**DOI:** 10.1038/srep18883

**Published:** 2015-12-22

**Authors:** Jinping Xu, Jiaojian Wang, Lingzhong Fan, Hai Li, Wen Zhang, Qingmao Hu, Tianzi Jiang

**Affiliations:** 1 Institute of Biomedical and Health Engineering, Shenzhen Institutes of Advanced Technology, Chinese Academy of Sciences, Shenzhen 518055, China; 2 Key Laboratory for NeuroInformation of the Ministry of Education, School of Life Science and Technology, University of Electronic Science and Technology of China, Chengdu 625014, China; 3 Brainnetome Center, Institute of Automation, Chinese Academy of Sciences, Beijing 100190, China; 4 National Laboratory of Pattern Recognition, Institute of Automation, Chinese Academy of Sciences, Beijing 100190, China; 5 CAS Center for Excellence in Brain Science, Institute of Automation, Chinese Academy of Sciences, Beijing 100190, China; 6 The Queensland Brain Institute, University of Queensland, Brisbane, QLD 4072, Australia

## Abstract

The middle temporal gyrus (MTG) participates in a variety of functions, suggesting the existence of distinct functional subregions. In order to further delineate the functions of this brain area, we parcellated the MTG based on its distinct anatomical connectivity profiles and identified four distinct subregions, including the anterior (aMTG), middle (mMTG), posterior (pMTG), and sulcus (sMTG). Both the anatomical connectivity patterns and the resting-state functional connectivity patterns revealed distinct connectivity profiles for each subregion. The aMTG was primarily involved in the default mode network, sound recognition, and semantic retrieval. The mMTG was predominantly involved in the semantic memory and semantic control networks. The pMTG seems to be a part of the traditional sensory language area. The sMTG appears to be associated with decoding gaze direction and intelligible speech. Interestingly, the functional connectivity with Brodmann’s Area (BA) 40, BA 44, and BA 45 gradually increased from the anterior to the posterior MTG, a finding which indicated functional topographical organization as well as implying that language processing is functionally segregated in the MTG. These proposed subdivisions of the MTG and its functions contribute to understanding the complex functions of the MTG at the subregional level.

The middle temporal gyrus (MTG), which is bounded dorsally by the superior temporal gyrus (STG) and ventrally by the inferior temporal gyrus (ITG), is a brain region which is unique to human beings, with no homology in non-human primates^1^. Unlike the STG, which has been confirmed to play an important part in auditory perception, or the ITG, which has been identified as the location of higher levels of the ventral visual processing stream, less is known about the functions of the MTG. Many studies have investigated the functional roles of the MTG and have reported that, in addition to involvement in language processing^2^,^3^,^4^,^5^,^6^,^7^, the MTG is involved in many other functions, such as observation of motion^8^, deductive reasoning^9^, and dynamic facial expressions^10^. Although the MTG has been found to be associated with various functions, the functional organization of this area, especially determining whether the anterior or posterior MTG serves as a semantic hub, remains controversial^11^,^12^. In addition, a meta-analysis found that the MTG was involved in two well-known networks, the default mode (DMN) and semantic memory (SM) networks^13^. However, the precise arrangement of these functions within the MTG is unclear. Given its complex and diverse functions, functionally distinctive subregions may exist in the human MTG. Therefore, obtaining a parcellation scheme for the MTG that is consistent with previous findings and identifying its connections and functions at the subregional level could greatly advance studies in this area.

Although a previous study regarded the MTG as a cytoarchitectonically homogeneous region^14^, several parcellation schemes for the MTG also exist. Based on differences in its myeloarchitectonic properties, the left MTG was subdivided into 6 different subregions^15^. Subsequently, another study identified three subregions from the anterior to the posterior of the MTG based on anatomical observations of the sulci or gyri^16^, and a topographic landmark-based mapping study further showed a similar subdivision of the MTG^17^. However, these subdivisions of the MTG, based on post-mortem analyses of myelo- or cyto-architectonic information or planes and sulci information, can only reflect the internal microstructure of a brain area but not the connectivity with other brain areas.

It has been well established that the functional segregation of the brain can be characterized by its external connectivity patterns^18^. Thus, defining the MTG subregions by identifying their anatomical connectivity patterns could better characterize the anatomical and functional organization of this area. Moreover, recent advances in diffusion magnetic resonance imaging have made it possible to show inter-regional anatomical connectivity which has been widely used to define the functional subregions of the brain. Based on their distinct *in vivo* connectivity patterns, the thalamus was firstly parcellated into component subregions by Behrens *et al.*^19^. Subsequently, many cortical areas, such as cingulated cortex^20^, parietal cortex^21^,^22^,^23^, superior frontal gyrus^24^, frontal pole^25^, and temporal pole^26^, have been parcellated to define the functional subregions. These findings have been proved to be consistent with those from cytoarchitecture-based studies. Thus, we adopted the same approach to parcellate the MTG into the functional subregions and characterize its functional organizations.

Because homology of the human MTG does not exist, we do not have data on the connectivity patterns of the MTG from non-human primate studies. Recently, Binney *et al.*^1^ showed three main white matter tracts connecting the temporal lobe to the frontal-parietal language network. Detailed white matter connectivity and the functions of the human MTG that are associated with this connectivity have the potential to provide new insight into its distinct functions. Specifically, exploring how the MTG connects with other brain regions at the subregional level may facilitate the understanding of how language is processed.

In our study, we first subdivided the MTG into subregions based on its various anatomical connectivity profiles. Then, we delineated the functional and anatomical connectivity patterns of each subregion by mapping the whole brain anatomical connectivity and resting-state FC patterns. Finally, we explored the potential language-related functions in which each subregion participates by comparing the resting-state FCs between each subregion of the MTG and the frontal-parietal language areas with each of the others.

## Results

### Tractography-based parcellation of the bilateral MTG

**Figure 1:**
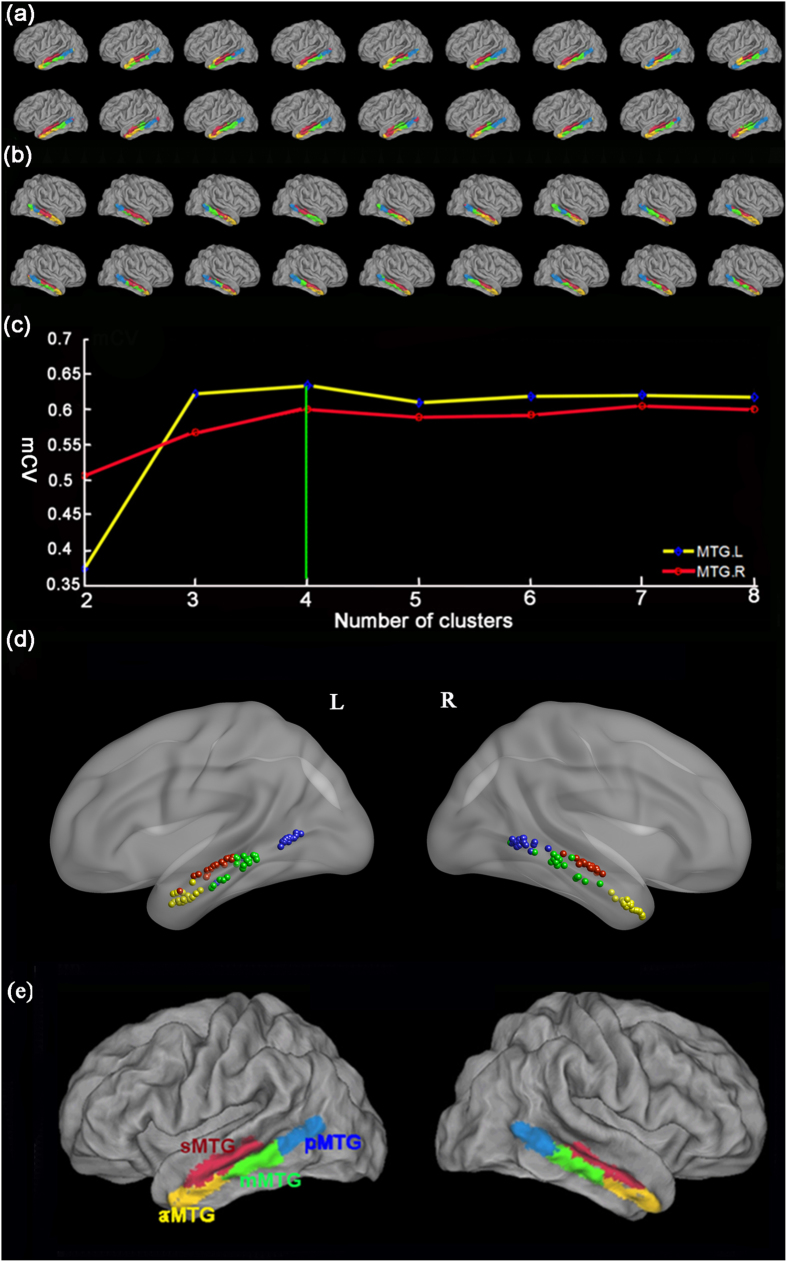
Results of the tractography-based parcellation of the bilateral middle temporal gyrus (MTG). The connectivity-based parcellation results of the left MTG (**a**) and right MTG (**b**) of all the 18 participants are shown using the PALS template with Caret software (http://brainmap.wustl.edu). The anterior subregion was colored with yellow, middle subregion colored with green, posterior subregion colored with blue, and sulcus subregion colored with red. (**c**) Average Cramer’s V was used to determine the consistency of parcellation results of bilateral MTGs for k = 2, 3, …, 8 clusters (blue: left MTG; red: right MTG). Cramer’s V ranges from 0 to 1 with higher values indicating good consistency. The four-cluster solution had the highest Cramer’s V for the bilateral MTG and was selected for further analyses. (**d**) Distribution of the centroid of each cluster by computing the mean x, y, and z coordinates in all the 18 subjects. All the locations were visualized with the BrainNet Viewer software (http://www.nitrc.org/projects/bnv/). The centroid of each cluster in the 18 individual’s brains was gathering into 4 clusters and their distribution pattern was similar with the maximum probabilistic map. (**e**) The maximum probabilistic map of MTG overlaid on a structural MNI brain using Caret software. The human MTG was reproducibly subdivided into anterior (yellow), middle (green), posterior (blue), and sulcus (red) subregions. Abbreviations: L, left, R, right, mCV, Average Cramer’s V, aMTG, anterior part of the middle temporal gyrus, mMTG, middle part of the middle temporal gyrus, pMTG, posterior part of the middle temporal gyrus, and sMTG, sulcus of the middle temporal gyrus.

Using *in vivo* probabilistic tractography and a spectral clustering algorithm, the MTGs were separately subdivided into 2–8 distinct areas with various connectivity patterns in the individual space for each subject. The maps of 4 distinct areas of the left MTG for each participant are shown in Fig. 1a and those of the right MTG in Fig. 1b.

To select the optimum number of clusters, the consistency characterized by Cramer’s V^24^ between each pair of subjects was calculated for k = 2, 3… 8. Thus, the four-way parcellation of left and right MTGs with the highest mean Cramer’s V values across all the subjects were selected for further analyses (Fig. 1c). Then, a stability map was made by computing the centroid of each cluster (mean x, y, and z coordinates) across subjects to further evaluate the consistency (Fig. 1d). Finally, the maximum probability maps of bilaterally MTGs were computed (Fig. 1e).

### Anatomical connectivity patterns of the MTG subregions

**Figure 2:**
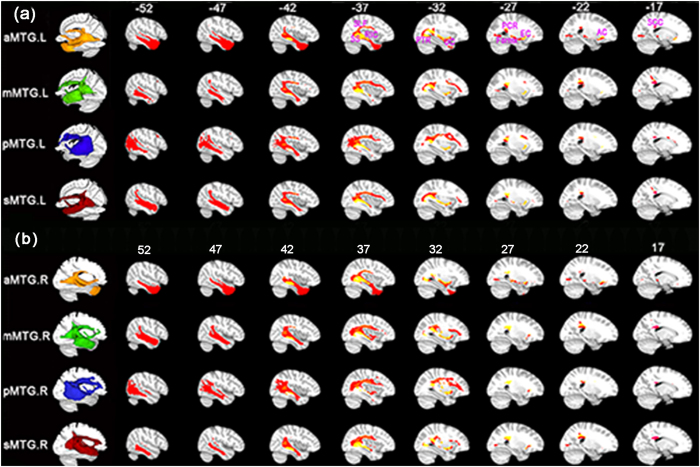
Population maps of the probabilistic tractography patterns. (**a**) for the left aMTG, mMTG, pMTG, and sMTG subregion, and (**b**) for the right MTGs. The fibers in the first column are shown on the ICBM152 template in the MNI space with Mango (http://www.brainmap.org) and multi-slice views are shown with MRIcron. To obtain the population maps, we first performed the whole brain probabilistic fiber tracking seeded in the aMTG, mMTG, pMTG, and sMTG. Second, we calculated the connection probability and thresholded it at p > 0.04% at individual level. Third, the fiber connections of each subject were binarized and warped into standard MNI space. Forth, we obtained a population map by averaging the binarized maps across all the 18 subjects. Fifth, we set a threshold at 50% in the population map to identify the main white matter pathway of each MTG subregion. Finally, we used JHU white matter tractography atlas to label the main fiber pathways. According to the JHU atlas, the main fiber tracts are illustrated and marked in yellow to orange, including the superior longitudinal fasciculus (SLF), sagittal stratum (SS), posterior thalamic radiation (PTR), external capsule (EC), posterior corona radiata (PCR), fornix (Fornix), anterior corona radiata (ACR), splenium of corpus callosum (SCC), retrolenticular part of internal capsule (RIC), uncinate fasciculus (UF).

The anatomical connectivity pattern of each MTG subregion is illustrated in Fig. 2. We used the Johns Hopkins University (JHU) white matter tractography atlas to label the main fiber pathways^27^. Several fiber pathways were labeled, including the superior longitudinal fasciculus (SLF), inferior frontal-occipital fasciculus (IFOF), uncinate fasciculus (UF), posterior thalamic radiation (PTR), external capsule (EC), retrolenticular part of internal capsule (RIC), fornix, anterior corona radiate (ATR), posterior corona radiate (PTR), and splenium of the corpus callosum (SCC). Most of these fibers were connected between the MTG and the frontal-parietal language areas.

**Figure 3:**
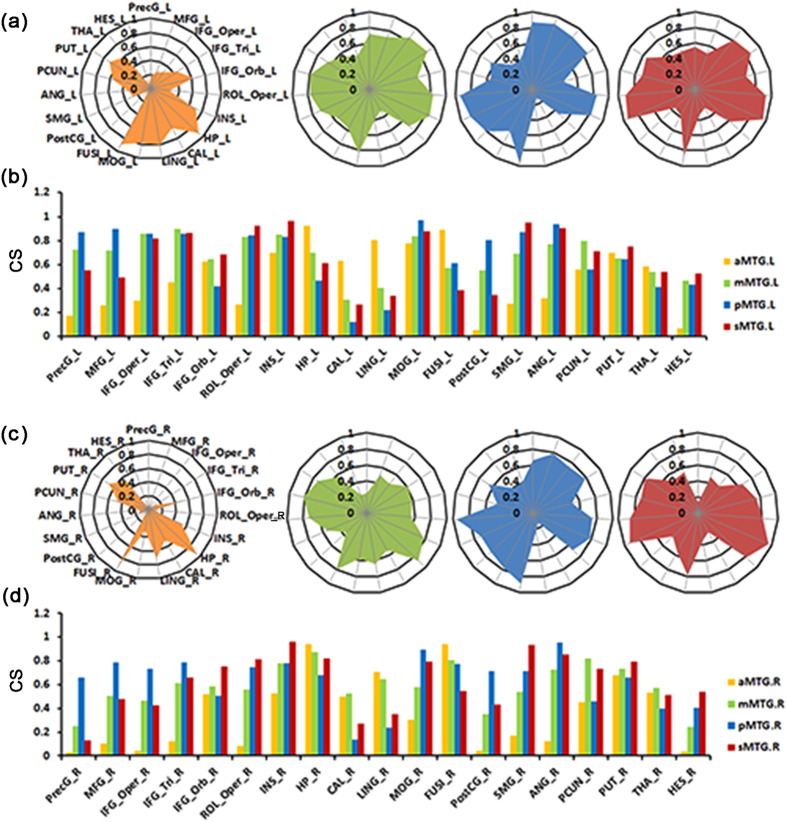
The average connectivity strengths (CS) between the bilateral MTG subregions and target regions ((a) and (b) for the left, (c) and (d) for the right). The average connectivity strength of each MTG subregion with 41 AAL regions is calculated within the same hemisphere. All regions which had a value above 0.5 with any MTG subregions are shown in the bar graphs, resulting in 19 brain targets. Abbreviations: ANG, angular gyrus; CAL, calcarine; FUSI, fusiform; HES, Heschl gyrus; HP, hippocampus; IFG_Oper, opercular parts of the inferior frontal gyrus; IFG_Orb, orbital part of the inferior frontal gyrus; IFG_Tri, triangular parts of the inferior frontal gyrus; INS, insula; LING, lingual gyrus; MFG, middle frontal gyrus; MOG, middle occipital gyrus; PCUN, precuneus; PostCG, postcentral gyrus; PreCG, precentral gyrus; PUT, putamen; ROL_Oper, rolandic operculum; SMG, supramarginal gyrus; and THA, thalamus.

The anatomical connectivity patterns between each MTG subregion and the 19 target brain regions in the ipsilateral hemisphere are shown in Fig. 3. The target brain areas were: the precentral gyrus (PreCG), middle frontal gyrus (MFG), opercular part of the inferior frontal gyrus (IFG_Oper), triangular part of the frontal gyrus (IFG_Tri), orbital part of the inferior frontal gyrus (IFG_Orb), rolandic operculum (ROL_Oper), insula (INS), hippocampus (HP), calcarine (CAL), lingual gyrus (LING), middle occipital gyrus (MOG), fusiform gyrus (FUSI), postcentral gyrus (PostCG), supramarginal gyrus (SMG), angular gyrus (ANG), precuneus (PCUN), putamen (PUT), thalamus (THA), and Heschl’s gyrus (HES).

The anatomical connectivity profiles were similar in both hemispheres with several variations. Of the four MTG subregions in the left hemisphere, the aMTG showed strong connectivity (>0.6) with the IFG_Orb, INS, HP, CAL, LING, FUSI, and PUT. The mMTG showed strong connectivity (>0.6) with the PreCG, MFG, IFG_Oper, IFG_Tri, IFG_Orb, ROL_Oper, INS, HP, MOG, SMG, ANG, PCUN, and PUT. The pMTG showed strong connectivity (>0.6) with the PreCG, MFG, IFG_Oper, IFG_Tri, IFG_Orb, INS, MOG, PostCG, SMG, ANG, and PUT. The sMTG showed strong connectivity (>0.6) with the IFG_Oper, IFG_Tri, IFG_Orb, ROL_Oper, INS, HP, MOG, SMG, ANG, PCUN, and PUT. We found some variations in the anatomical connectivity profiles of the right MTG subregions as compared with the left. First, the right aMTG showed strong connectivity (>0.6) with fewer regions than did the left aMTG, in that the right aMTG only showed connectivity with the HP, LING, FUSI and PUT. Additionally, the right mMTG showed strong connectivity (>0.6) with the FUSI and LING but not with the PreCG, MFG, IFG_Oper, IFG_Orb, ROL_Oper, and HP.

### Whole brain resting-state functional connectivity pattern

**Figure 4:**
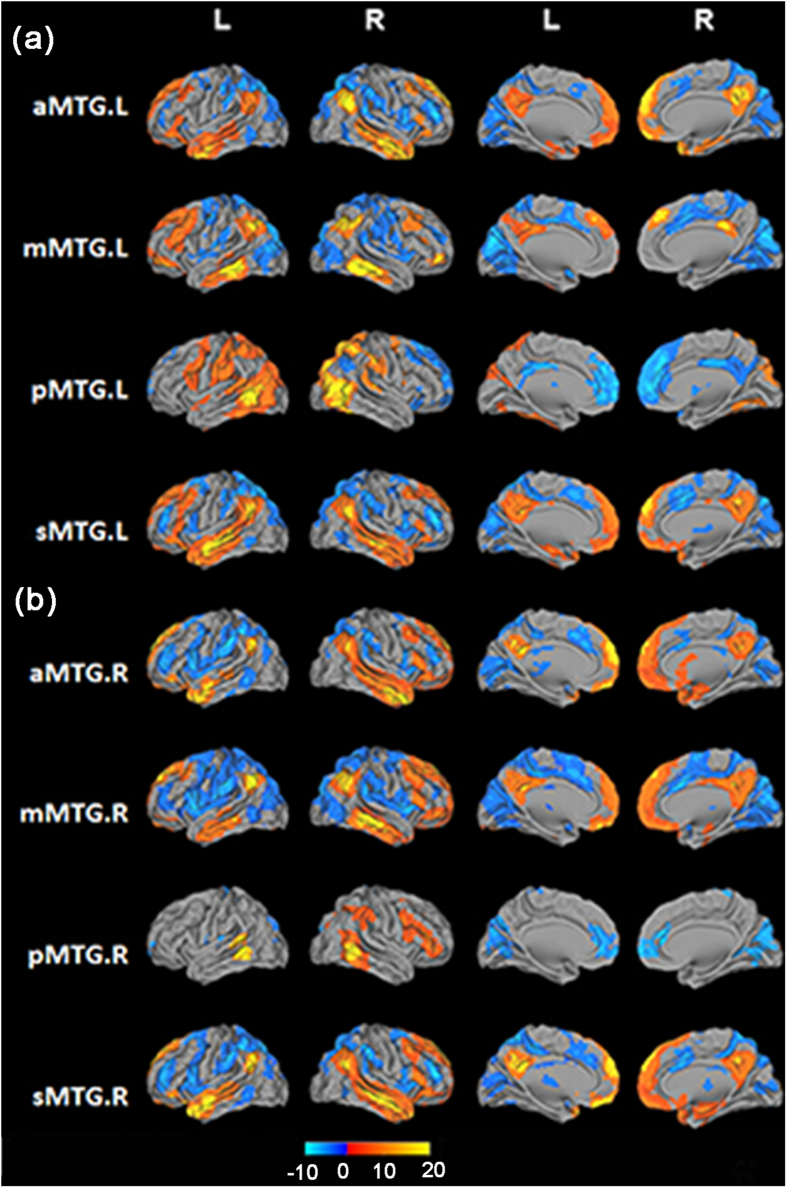
Resting-state functional connectivity (FC) patterns for the bilateral aMTG, mMTG, pMTG, and sMTG ((a) for the left MTG and (b) for the right MTG). All results are displayed using a voxel-level statistical threshold of *p* < 0.01 corrected for the false discovery rate (FDR) with a cluster threshold of 100 voxels. The resting-state FC profiles are mapped to a 3D brain surface with Caret software.

Functional connectivity networks for each MTG subregion, which could indicate the variety of functional roles played by each subregion, were systematically delineated using resting-state FC analyses. The resting-state FC results (not including the cerebellum) for each MTG subregion are displayed in Fig. 4.

#### Positively correlated networks

From the resting-state FC pattern for each subregion, we found that the aMTG was positively correlated with the anterior part of the superior and inferior temporal gyri, temporal pole, parahippocampus gyrus, orbital part of inferior frontal gyrus, and areas of the DMN, including the medial superior frontal cortex, medial orbito-frontal gyrus, posterior cingulate cortex, posterior temporoparietal junction, and precuneus. The mMTG was primarily correlated with the inferior temporal gyrus, angular gyrus, supramarginal gyrus, middle frontal gyrus, medial superior frontal cortex, triangular and orbital part of inferior frontal gyri, and posterior cingulate gyrus. The pMTG was positively correlated with the posterior part of the superior and inferior temporal gyri, supramarginal gyrus, middle frontal gyrus, opercular part of inferior frontal gyrus, fusiform gyrus, pre- and post-central gyri, middle occipital gyrus, and superior and middle parietal lobule. The functional connectivity pattern of the sMTG was similar to that of the aMTG, except that the regions that were positively correlated with the sMTG included the middle cingulate gyrus but not the parahippocampus gyrus.

#### Negatively correlated networks

The aMTG was primarily negatively correlated with the posterior inferior temporal gyrus, middle frontal gyrus, the triangular part of inferior frontal gyrus, supplement motor areas, middle cingulate gyrus, and calcarine. The mMTG was primarily negatively correlated with the cuneus, precuneus, supplement motor areas, calcarine, middle cingulate gyrus, opercular part of the inferior frontal gyrus, and insula. The pMTG was primarily negatively correlated with the anterior and middle cingulate gyri, angular gyrus, and areas of the DMN, including the medial superior frontal cortex and posterior cingulate cortex. The sMTG was primarily negatively correlated with the posterior inferior temporal gyrus, orbital part of superior and middle frontal gyri, middle frontal gyrus, and triangular part of the inferior frontal gyrus, supplement motor areas, middle cingulate gyrus, and calcarine.

In addition, the bilateral MTG subregions showed similar functional connectivity patters for both the positively and negatively correlated brain regions except for the following: (1) the right mMTG showed strong positive correlations with the DMN areas, and (2) the right pMTG showed less positive correlations with the parietal lobule. Thus, the distinct positively and negatively correlated networks that we observed may reflect competition between neuronal activities. Moreover, reversed functional connections between the MTG subregions and the DMN areas/the pre- central gyrus/post-central gyrus were found. Specifically, the aMTG showed positive correlations with the DMN regions whereas the pMTG showed negative correlations. Moreover, the pMTG showed positive correlations with the pre- and post-central gyrus while the other three MTG subregions showed negative correlations.

### Resting-state functional connectivity with language areas

**Figure 5:**
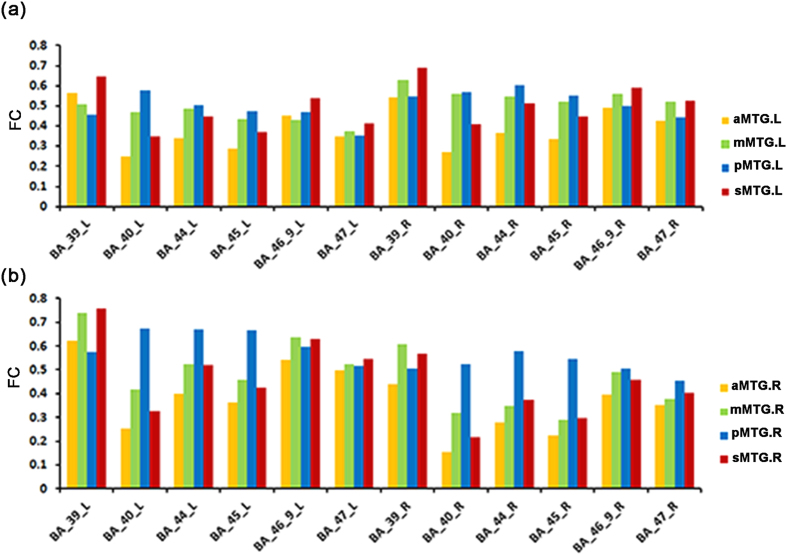
Bar graphs of the average resting-state FC between the bilateral MTG subregions and 6 language regions in the Broadman template ((a) for the left MTG and (b) for the right MTG). Abbreviations: BA_39, Broadman area 39; BA_40, Broadman area 40; BA_44, Broadman area 44; BA_45, Broadman area 45; BA_46_9, Broadman area 46 and 9; and BA_47, Broadman area 47. sMTG (red), aMTG (yellow), mMTG (green), and pMTG (blue).

The resting-state FC patterns between each MTG subregion and language-related areas are shown in Fig. 5. Except for the sMTG (the red region in the Fig. 1e) which was located in the sulcus of the MTG, the other three MTG subregions showed a gradually increasing functional connectivity pattern with BA 40, BA 44, and BA 45 from the anterior to the posterior MTG. In addition, all MTG subregions showed a higher FC with the ipsilateral BA 39, BA 46_9, and BA 47.

## Discussion

In this study, we subdivided the bilateral MTGs using a tractography-based parcellation scheme based on inter-regional anatomical connection profiles. The validity and reliability of this method have been extensively confirmed in previous parcellation studies^21^,^24^,^25^,^26^. Four distinct subregions in the human MTG including the aMTG, mMTG, pMTG, and sMTG were identified. It was also found that the MTG subregions had its specific anatomical and functional connectivity patterns, and these subregions were involved in different functional networks.

In comparison with the myeloarchitecture-based mapping of the temporal cortex^15^, the aMTG primarily corresponded to area tmag.d.aif, and the mMTG primarily corresponded to tmag.d.md, whereas the pMTG predominantly corresponded to tmag.d.s and tmag.d.p*. Furthermore, we also found that the aMTG in our work corresponded approximately to rostral portions of the MTG (rMTG) and that the mMTG and pMTG were consistent with the caudal portions of the MTG (cMTG) identified by Yate *et al.*^28^. Despite the differences, the parcellation of the MTG in our study was similar to that reported in the study by Rademacher and his colleagues^16^, which identified 3 subregions from the anterior to the posterior MTG. Specifically, the aMTG in our study approximately corresponded to the anterior part of the MTG (T2a), the mMTG to the posterior part of the MTG (T2p), and the pMTG to the anterior part of the temporoocciptal part of the MTG (TO2). Moreover, our parcellation results were also similar to those from Kim and his colleagues’ study^17^ which was based on cytoarchitecture, in that their rostral portions of the MTG (rMTG) correspond approximately to our aMTG, their intermediate portions (iMTG) to our mMTG, and their caudal portions of the MTG (cMTG) to the pMTG in our work. These consistent parcellation results, which were based on different properties, may indicate correspondence between the cytoarchitecture, which reflects the detailed inner organization of the cortical areas, and the connective architecture, which determines the functions of this area. However, because of the extent of the variability in the human MTG and differences in the methodologies used and their inherent limitations, a direct comparison of the *in vivo* parcellation results with previous cytoarchitectonic or myeloarchitectonic findings should nevertheless be performed with caution, and inconsistencies between these studies should be expected as well.

The whole brain anatomical connectivity patterns for each MTG subregion were similar to Binney and his colleagues’ study^1^, which showed three well-known association tracts connecting the temporal lobe to the fronto-parietal language network. Moreover, the three fibers could be tracked from all the MTG subregions in our anatomical connectivity patterns, which may indicate that they are not singular bundles but have various branches connecting different areas. This was similar to Fan and his colleagues’ study^26^ which also found that the UF could be tracked from all the temporal pole (TP) subregions and confirmed that there were two sub-components of the UF with differential connectivities between different TP subregions and different frontal-limbic areas. In addition to finding similarities, the following differences were also identified in the anatomical connectivity patterns. First, the anterior corona radiate (ATR) was only observed in the anatomical connectivity patterns of the first three MTG regions. Second, the fornix was identified in the aMTG and sMTG. Third, the anatomical connectivity strength was confirmed as being significantly different by a quantified anatomical connectivity strength analysis.

By quantifying the anatomical connectivity strength and analyzing the resting-state FC patterns, a different functional network was identified for each MTG subregion. These results can not only provide a scheme for investigating the structural and functional characteristics of the MTG at the subregional level but may also further the structural and functional studies of the human MTG.

The resting-state FC patterns of the aMTG revealed that the aMTG was primarily connected with DMN-related brain regions, suggesting that the bilateral aMTG is a critical part of the DMN. Thus, previous identifications of the MTG in the DMN may be confined to the aMTG^29^. The anatomical connectivity mapping analyses revealed that the aMTG connected with the anterior frontal cortex via the UF through the rostral superior temporal gyrus and the temporal pole. Because the UF is considered to be a ventral limbic pathway which is important for sound recognition^30^, we concluded that the aMTG plays an important role in language comprehension^31^. This finding was consistent with the finding from Fan and his colleagues’ study which also found that the UF connected with the anterior temporal lobes, anterior middle temporal gyrus, and dorsal temporal pole^26^. In comparison with the three other MTG subregions, the aMTG showed stronger anatomical connectivity with the medial temporal lobe, including the hippocampus, lingual gyrus, and fusiform gyrus. This was further confirmed by the whole resting-state FC patterns of the aMTG. A previous study demonstrated that stronger activations of these regions in children were closely correlated with higher retrieval fluency, suggesting that the aMTG might play a key role in semantic retrieval^32^.

The mMTG was predominantly correlated with the medial superior frontal cortex, posterior cingulate cortex, temporoparietal junction, precuneus, and the triangular and orbital parts of the inferior frontal gyrus. Those areas have been found to be critical for semantic memory (SM). This finding was further supported by our quantitative anatomical connectivity analysis, which showed stronger anatomical connectivity between the mMTG and the precuneus as compared to the other three MTG subregions and indicated that the previously identified anterior MTG in the SM is confined to the mMTG. Although previous studies showed that the SM and the DMN network overlap spatially^13^, recent studies have indicated that portions of the anterior temporal lobe, including the mMTG, play an important role in representing and retrieving social knowledge, which is a specific type of semantic memory^32^. In addition, the connectivity of the pMTG with the triangular portions of the inferior frontal gyrus, the orbital portions of the inferior frontal gyrus, and the angular gyrus was consistent with a previous task-based semantic study, which found that these areas were co-activated in a high versus low semantic control demands conjunction^4^. The functional specialization of the mMTG was also confirmed by whole brain anatomical connectivity pattern analyses which showed direct pathways between the mMTG and the inferior frontal gyrus.

The pMTG was primarily located at the posterior part of the MTG. From the resting-state FC patterns, we found that the pMTG is primarily connected with the opercular portion of the inferior frontal gyrus, premotor cortex, pre- and post-central gyrus, fusiform gyrus, supramarginal gyrus, and middle occipital gyrus. In addition, the pMTG also showed strong anatomical connectivity with the middle frontal gyrus, triangular part of the inferior frontal gyrus, rolandic operculum, and angular gyrus. Both the resting-state FC patterns and the anatomical connectivity pattern implied that the pMTG plays an important role in language processing, especially in language repetition or reading^33^. This hypothesis was supported by previous task-based fMRI studies, which showed that the pMTG co-activated with the opercular part of the inferior frontal gyrus, premotor cortex, pre- and post-central gyri, and fusiform gyrus in a controlled word task, and co-activated with the middle frontal gyrus, pre-supplementary motor area, and middle occipital gyrus in a verb processing task^34^. Moreover, the specific connectivity patterns of this area were also consistent with previous findings which suggested that the pMTG might be related to the semantic processing of visually presented words and affective prosody^35^,^36^. However, the pMTG showed weaker connectivity with the thalamus than the other three anterior MTG subregions, which showed stronger connectivity with the thalamus in both brain hemispheres. This finding was supported by a previous anatomical connectivity study of the thalamus^19^, which revealed that fibers from voxels in the mediodorsal nucleus extended at first posteriorly around the posterior edge of the thalamus and then anteriorly to the anterior temporal cortex, including the anterior part of the MTG.

The sMTG, which was primarily located at the sulcus of the middle temporal gyrus, corresponded to the aSTS. Research in macaque neurophysiology suggested that the aSTS plays a notable role in representing the perceived direction of another’s social attention cues, as conveyed by head orientation, gaze direction, and body posture^37^,^38^,^39^. A similar function was found in humans in that the aSTS, together with the precuneus, was involved in perceptual coding of another’s eye gaze direction^40^. Additionally, the aSTS, together the medial prefrontal cortex, was involved in the experience of joint attention with another individual^41^. Our functional connectivity pattern for the sMTG also showed positive correlations with both the medial prefrontal cortex and the precuneus. This finding indicated that the sMTG was associated with decoding gaze direction. In addition, many functional imaging studies^42^,^43^,^44^ have concluded that the aSTS responds preferentially to intelligible speech, including acoustic-phonetic, semantic, and syntactic processing, as well as their associated representations. This conclusion was supported by our functional connectivity pattern of the sMTG which showed a positive correlation with several SM-related regions and, thus, may be seen as emphasizing an important role that the sMTG may play in intelligible speech. The anatomical connectivity pattern of the sMTG showed strong connectivity with the supramarginal gyrus, rolandic operculum, and Heschl’s gyrus. Those regions were co-activated in two different kinds of function sessions that used verbalization during mental arithmetic^45^. Such connectivity of the sMTG suggested that this area might also be involved in a verbal cognitive style of mental arithmetic, which is known to be an aspect of high cognitive functioning. In addition to these cortical connection patterns, the sMTG also showed differences in sub-cortical connection patterns from the anatomical connectivity patterns. The sMTG showed strong connectivity with the insula and putamen, whereas the other three MTG subregions showed less connectivity strength with these areas in both brain hemispheres. This effect was possibly because the sMTG is located next to the medial superior temporal gyrus, which is known to be connected with frontal areas through the inferior fronto-occipital fasciculus extending directly to the putamen and insula via the external capsule^46^,^47^. Furthermore, the whole brain anatomical connectivity results from the sMTG indicated that the major anatomical pathways between the MTG and the putamen/the insula were primarily involved with the inferior fronto-occipital fasciculus and external capsule, a finding which was consistent with the white matter fasciculi work of Catani *et al.*^47^ and the ventral auditory-language pathways of Geoffrey *et al.*^46^.

Both the whole anatomical and FC patterns of the MTG subregions showed strong connectivity with the frontal-parietal language areas. Quantitative functional connectivity analyses with the frontal and parietal language areas revealed that the subregions in the MTG (aMTG, mMTG, pMTG) showed a gradual increase in functional connectivity strength with BA 40, BA 44, and BA 45 along a longitudinal axis from the anterior to the posterior MTG. The different connectivity patterns with the main language-related areas suggest that different subregions in the MTG play different roles in language processing^48^. Clinical studies of semantic dementia and positron emission tomography (PET)-based neuroimaging investigations in healthy subjects indicated that the anterior temporal lobes, including the aMTG, act as a semantic hub, which draws together visual, auditory, motor, functional and ‘encyclopedic’ knowledge about words and concepts to form high-level, amodal conceptual representations^49^,^50^,^51^. However, other studies employing voxel-based lesion-symptom mapping^12^ and transcranial magnetic stimulation (TMS)^52^ indicated that the pMTG acts as a semantic hub. Gregory and his partners^11^ proposed that the pMTG is at the core of several brain areas which are primarily involved in accessing lexical and semantic information. Our findings using FC analyses with specific language related brain areas showed that the pMTG had strong connections with language-related areas and suggested that the pMTG may act as a semantic hub. Moreover, previous studies^1^ demonstrated a direct pathway that is predominately associated with the pMTG and the opercular- and triangular part of the inferior frontal gyrus (BA 44 and BA 45, known as Broca’s region). This pathway appeared to be mostly attributable to the arcuate fasciculus (AF), by which language information is transported^1^,^53^. Moreover, the pMTG also exhibited pathways to the supramarginal gyri (BA 40) via fibers that belong to either the AF or the middle longitudinal fasciculus (MDLF)^1^. However, due to the absence of homology of the human MTG with non-human primates, no evidence is available from the comparative neurologic literature of non-human primates.

There are several limitations in our current study. First, considerable stability of the four-way parcellation of MTGs was found across all the 18 subjects, the accuracy and validity of the parcellation results need to be further verified. Second, in our current study, we used probabilistic tractography to map the anatomical connectivity patterns which may be influenced by many factors, such as the size of mask, the distance and the geometry of the pathways. Additionally, the traditional DTI method has its inherent limitations to accurately characterize fiber directions, especially to distinguish fibers which run in parallel but belong to different tracts^54^. Thus, more plausible methods are yet to be developed to more accurately characterize the anatomical connections of the human brain.

To the best of our knowledge, this is the first study to parcellate the human MTG based on its anatomical connectivity profiles using probabilistic tractography or to explore the anatomical and functional connectivity patterns at the subregional level.We demonstrated four distinct subregions in the bilateral MTG, the aMTG, mMTG, pMTG, and sMTG. By mapping the whole-brain anatomical connectivity patterns, elucidating the functional connectivity patterns, and performing a quantitative analysis of the anatomical connectivity strength for each MTG subregion, we found different functional networks with which they were involved. In addition, a gradual increase in the FC of the MTG subregions with language areas from the anterior to the posterior MTG subregions was identified. This finding suggested a topographical language functional organization in the MTG and might broaden our understanding of how MTG was involved in the processing of language information. The subregions can be further used as seed regions for comparing functional connectivity or structural connectivity in aphasia patients to reveal the neuropathological basis. Furthermore, this study to find different anatomical and functional connectivity patterns at subregional level can extend future post-mortem studies in this area.

## Materials and Methods

### Participants

The MRI data used in our study was accessed from the Human Connectome Project (HCP, http://humanconnectome.org/) dataset. The MRI data for the diffusion tensor imaging (DTI) included 18 healthy, right-handed subjects (9 males and 9 females, age range: 22–30 years), and those for the functional magnetic resonance imaging (fMRI) included 40 subjects (18 males and 22 females, age range: 22–35 years). None of the participants had ever suffered from any psychiatric or neurological disease, and none had any contraindications for MRI scanning. All participants signed an informed consent prior to participation in the study. All protocols were approved by the Ethics Committee of the Institute of Automation Chinese Academy of Sciences Review Board. All methods were carried out in accordance with approved guidelines and regulations.

### MRI data acquisition

DTI, structural MRI, and resting-state fMRI data were all collected using a HCP 3T Siemens Skyra scanner with a 32-channel head coil. Detailed data acquisition procedures and information can be found in the HCP scan protocols (http://humanconnectome.org/documentation/data-release/scan-protocols.html) and published papers from their team^55^,^56^. The DTI scheme contained a collection of 3 different gradient tables (b = 1000, 2000, and 3000 s/mm^2^) and each table was acquired once with right-to-left and left-to-right phase encoding polarities with 90 non-collinear diffusion gradients and 6 non-diffusion-weighted images (b = 0 s/mm^2^) using an HCP-specific variant of the multiband diffusion sequence (http://www.cmrr.umn.edu/multiband). From each participant, 111 slices were collected with a field of view (FOV) =210 × 180 mm^2^, flip angle (FA) =90°, echo time (TE) =89.50 ms, repetition time (TR) =5520 ms, and slice thickness = 1.25 mm, with no gap. This method resulted in voxel-dimensions of 1.25 × 1.25 × 1.25 mm^3^. Resting-state fMRI data was obtained using the following imaging parameters: TR/TE = 720/33 ms, FOV = 208 × 180 mm^2^, matrix = 104 × 90, FA = 52°, slice thickness = 2 mm, 72 slices covering the entire brain, and 1200 volumes. We also acquired averages of the two separate T1-weighted image sets, which were obtained using 0.7 mm isotropic resolution, TR/TE = 2400/2.14 ms, inversion time (TI) =1000 ms, FA = 8°, band width (BW) =210 Hz per pixel, echo spacing (ES) =7.6 ms, FOV = 224 × 180 mm^2^, matrix = 320 × 256, and sagittal slices.

### DTI data preprocessing

DTI data preprocessing included the following steps. First, the intensity of mean b0 image across the six diffusion series was normalized using the “topup” tool in FMRIB’s Diffusion Toolbox (FSL5.0; http://www.fmrib.ox.ac.uk/fsl). Then, distortions in the diffusion-weighted images caused by eddy currents and simple head motions were corrected using the “eddy” tool in FSL5. Finally, the transform between native diffusion space and native structural space was calculated. T1-weighted images from each subject were co-registered to the subject’s mean b0 image using a statistical parametric mapping package (SPM 8; http://www.fil.ion.ucl.ac.uk/spm). This resulted in a set of co-registered T1 images (rT1) in DTI space. Then, the T1 images obtained in diffusion space were transformed to Montreal Neurological Institute (MNI) space (HCP40_MNI_1mm.nii in the dataset). For more information, one can refer to “The Minimal Preprocessing Pipelines for the Human Connectome Project”^55^.

### Resting-state fMRI data preprocessing

The resting-state fMRI data was preprocessed using the dataset in MNINonLinear/Results/ that was provided by HCP after the data had passed through the fMRI volume pipeline, where it had already undergone motion correction, registration to standard space, grand mean scaling, and brain masking. The subsequent processing steps were: (1) temporal band-pass filtering (0.01-0.1Hz), (2) removing linear and quadratic trends, (3) full-width at half maximum (FWHM) spatial smoothing with a Gaussian kernel of 6 mm, (4) regressing out nuisance signals such as those from white matter (WM) and cerebrospinal fluid (CSF) as well as global signals and six motion parameters (However, the global signals were not regressed out in the resting-state fMRI data, which was used to calculate the FC between each MTG subregion and the language areas.), (5) normalizing the subjects’ time series datasets to percent change values in preparation for group analysis, and (6) resampling the functional data into a voxel size of 3 × 3 × 3 mm^3^ with the concatenated transformations. In the end, this procedure provided a four-dimensional residual time series in standard MNI space for each participant. All these proceeding steps used scripts provided by the 1000 Functional Connectomes Project (http://www.nitrc.org/projects/fcon_1000) with both FSL and AFNI software (http://www.afni.nim.nih.gov/).

### Definition of MTG seed masks

The bilateral MTG masks were defined using Freesurfer (http://surfer.nmr.mgh.harvard.edu/fswiki) and transformed from surface space to volume space. For the DTI analyses, the seed masks were transformed from MNI space back to individual native DTI space using an inverse linear transformation and nonlinear deformations in SPM8. Moreover, we checked the registration accuracy of each seed region on the coronal, axial, and sagittal planes slice-by-slice in native DTI space. For the resting-state FC analyses, the MTG seed masks were also resampled into 3 × 3 × 3 mm^3^ in MNI space.

### Definition of language brain regions

To explore the potential language-related functions in which each subregion participates, we defined a series of frontal and parietal language brain areas using the Brodmann template. These brain areas included BA 44 (pars opercularis), BA 45 (pars triangularis), BA 46 and BA 9 (dorsolateral prefrontal cortex), BA 47 (pars orbitalis), BA 39(angular gyrus), and BA 40 (supramarginal gyrus). These regions in MNI space were resampled into 3 × 3 × 3 mm^3^ for resting-state FC analyses.

### Probabilistic tractography

For the probability tractography of each seed mask, we performed the following steps in diffusion space using the FSL package: First, we used a multiple fiber extension from a previously-published diffusion modeling approach^19^ to calculate probability distributions for two fiber directions at each voxel in the MTG mask. Then, to estimate the connectivity probability, probabilistic tractography was applied by sampling 5000 streamline fibers per voxel in the MTG mask. For each sampled fiber, we first drew a sample direction from the local direction distribution at the seed voxel and then proceeded a fixed distance of 0.5 mm along this direction to a new position where we drew a new sample direction from the local distribution at this new position. This propagation procedure continued until the brain surface was reached or the path looped back on itself. In order to reduce the number of false positive connections, we thresholded the path distribution estimates with the connection probability value *p* < 0.002 (10 out of 5000 samples). Sequentially, the connection connectivity probability between voxels in the MTG and all of the remaining voxels in the brain were calculated and stored at a lower resolution of 5 × 5 × 5 mm^3^ to facilitate data storage^57^. Finally, a cross-correlation matrix was calculated based on the native connectivity matrix to quantify the similarity between the connectivity profiles of the MTG seed voxels^21^.

### Tractography-based parcellation

The cross-correlation matrix was then fed into spectral clustering for automated clustering to define different clusters^21^. We successfully obtained spatially contiguous subregions in the MTGs, although a few discontinuous voxels in the individual parcellation were identified. In order to avoid an arbitrary choice of the number of clusters, we used the any-two method in which any pairs of two subjects in the dataset were considered instead of the leave-one-out method to determine the number of clusters, which yielded optimal consistency across subjects. To achieve this, we first calculated the consistency between the parcellation result of each subject and any other subject in the dataset using Cramer’s V index^24^ for k = 2, 3, …, 8 clusters. Then, the values of Cramer’s V were averaged to obtain the average stability map. The Cramer’s V gives values within the interval [0, 1], within which high values indicate good consistency and a value of 1 indicates a perfect match. We then made a reliability map of the four-way parcellation results by computing the centroid of each cluster (mean x, y, and z coordinates) across subjects to evaluate the reliability.

We calculated the probabilistic maps in standard MNI space from the overlap of these clusters across subjects, producing a result which indicated the inter-individual variability of an area and represented the proportion of the population in which a cluster was present. In light of inter-individual differences in the MTG, we created maximum probability maps in standard MNI space for each MTG subregion to visualize.

### The anatomical connectivity patterns

To map each subregion’s whole brain anatomical connectivity pattern, we first performed the whole brain probabilistic fiber tracking by drawing 5000 samples from each voxel in the aMTG, mMTG, pMTG, and sMTG subregions to all the other voxels of the whole brain in the individual diffusion space. The MTG subregions were obtained by thresholding the probability map at 50% probability. Second, we calculated the connection probability between each subregion of the MTG and each other voxel of the brain. In order to reduce the false positive rates in fiber tracking, an individual level threshold for the connectivity probability at *p* > 0.04% (i.e., >2 of the 5000 samples generated from each seed voxel) was set in the diffusion space. Third, fiber connections of each subject were binarized and warped into standard MNI space. Forth, a population map for each MTG subregion was obtained by averaging the binarized maps across the 18 subjects. Fifth, we set a threshold at 50% in the population map (that is, displaying only those voxels that were present in at least 9 subjects) to identify the main WM pathway of each MTG subregion. Finally, we used JHU white matter tractography atlas to label the main fiber pathways.

Next, we employed the Automated Anatomical Labeling (AAL) atlas to subdivide the cerebral cortex and subcortical nuclei of each subject into different target areas (40 areas for each hemisphere, with the STG, MTG, IGT, the temporal pole, and cerebellum excluded). Then, for each subject, we ran probabilistic tractography by drawing 5000 samples from each voxel in the aMTG, mMTG, pMTG, and sMTG regions (thresholded at 50% probability) to the AAL regions in individual diffusion space and counted how many stream lines of random walks starting in each MTG subregion reached each target after thresholding 100 out of 5000 samples to reduce the false positives. The value, which was divided by the total number of voxels in the seed mask, was defined as the connection strength of each seed-target combination. Finally, we selected those target brain regions with a connection strength >0.5 in both hemispheres to map the anatomical connectivity fingerprints.

### The whole brain resting-state functional connectivity patterns

For each of the 40 subjects, the resting-state FC was defined as the Pearson correlation coefficients between the mean time series of each seed region and that of each voxel in the rest of the brain. Correlation coefficients were converted to *z* values using Fisher’s *z* transformation to improve normality. Next, a one-sample *t*-test was performed to identify voxels which showed significantly positive or negative correlations with the seed region in these normalized correlation maps. For all the above voxel-wise comparisons, the false discovery rate (FDR) method was used for multiple comparison correction (*p* < 0.01), and only clusters that contained a minimum of 100 voxels were reported.

### Resting-state functional connectivity with the language-related areas

All seed masks and target masks were first resampled to a resolution of 3 × 3 × 3 mm^3^ in standard MNI space. Then, we averaged all the time series of the seed voxels and target masks and calculated the Pearson correlation between each seed subregion in the MTGs and language-related regions. Finally, we calculated the average correlation coefficient across the 40 subjects.

## Additional Information

**How to cite this article**: Xu, J. *et al.* Tractography-based Parcellation of the Human Middle Temporal Gyrus. *Sci. Rep.* **5**, 18883; doi: 10.1038/srep18883 (2015).
